# Supplementary figures and images for: cotH Genes Are Necessary for Normal Spore Formation and Virulence in Mucor lusitanicus
Source: mBio. 2023 Jan 10;14(1):e03386-22. doi: 10.1128/mbio.03386-22 (PMC9973265; doi:10.1128/mbio.03386-22)

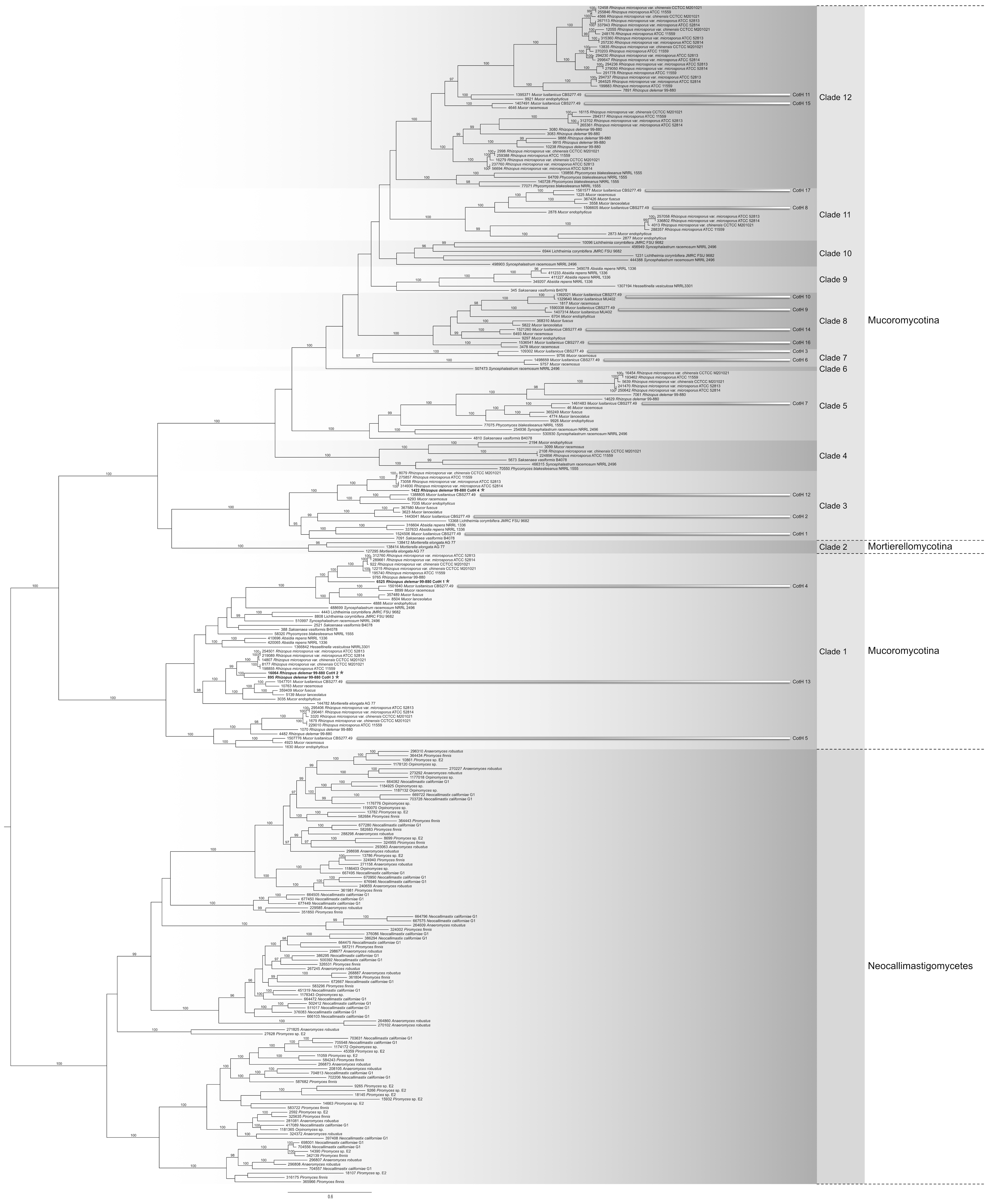

Supplement: FIG S1 [file mbio.03386-22-s0001.tif]

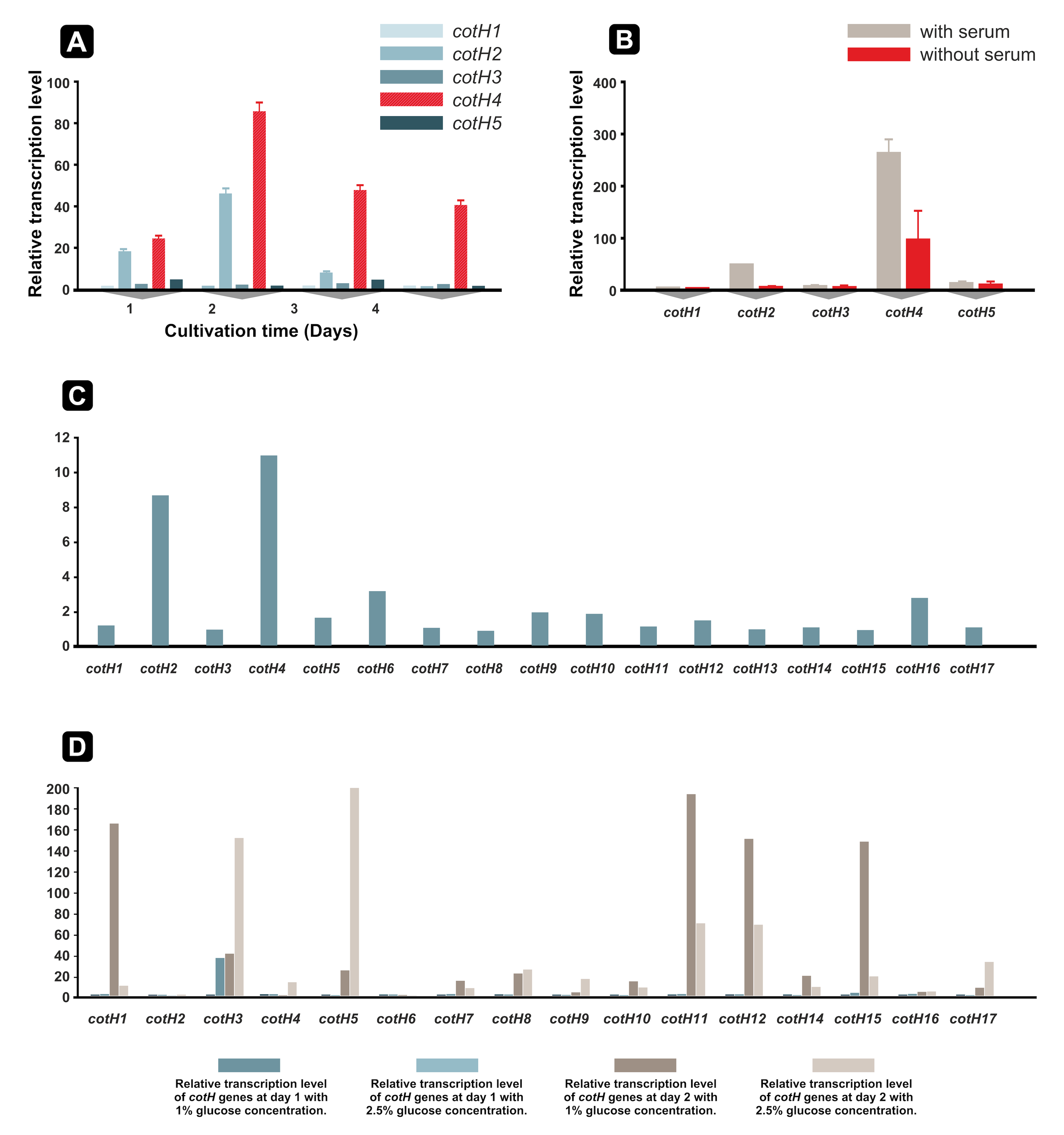

Supplement: FIG S2 [file mbio.03386-22-s0002.tif]

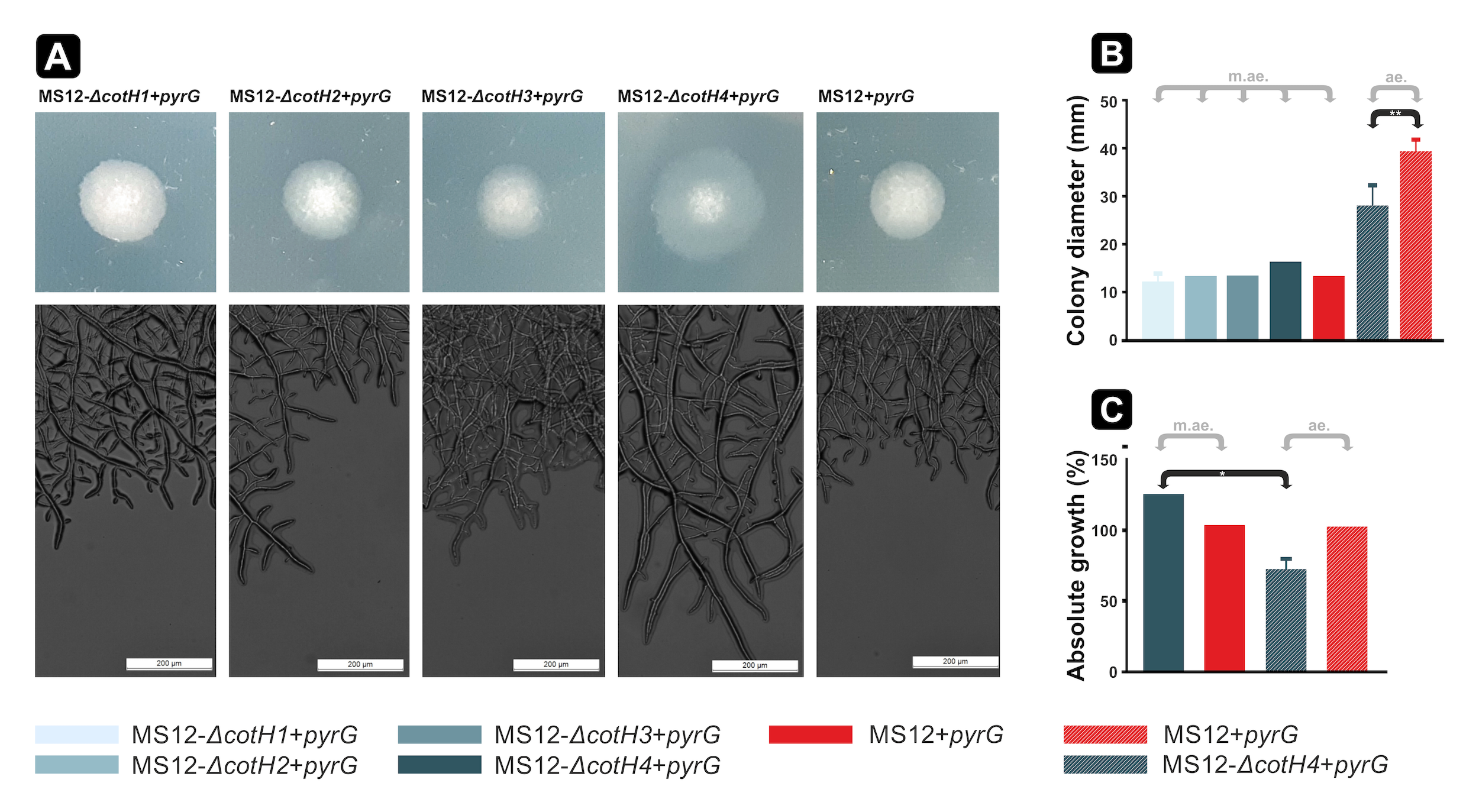

Supplement: FIG S3 [file mbio.03386-22-s0004.tif]

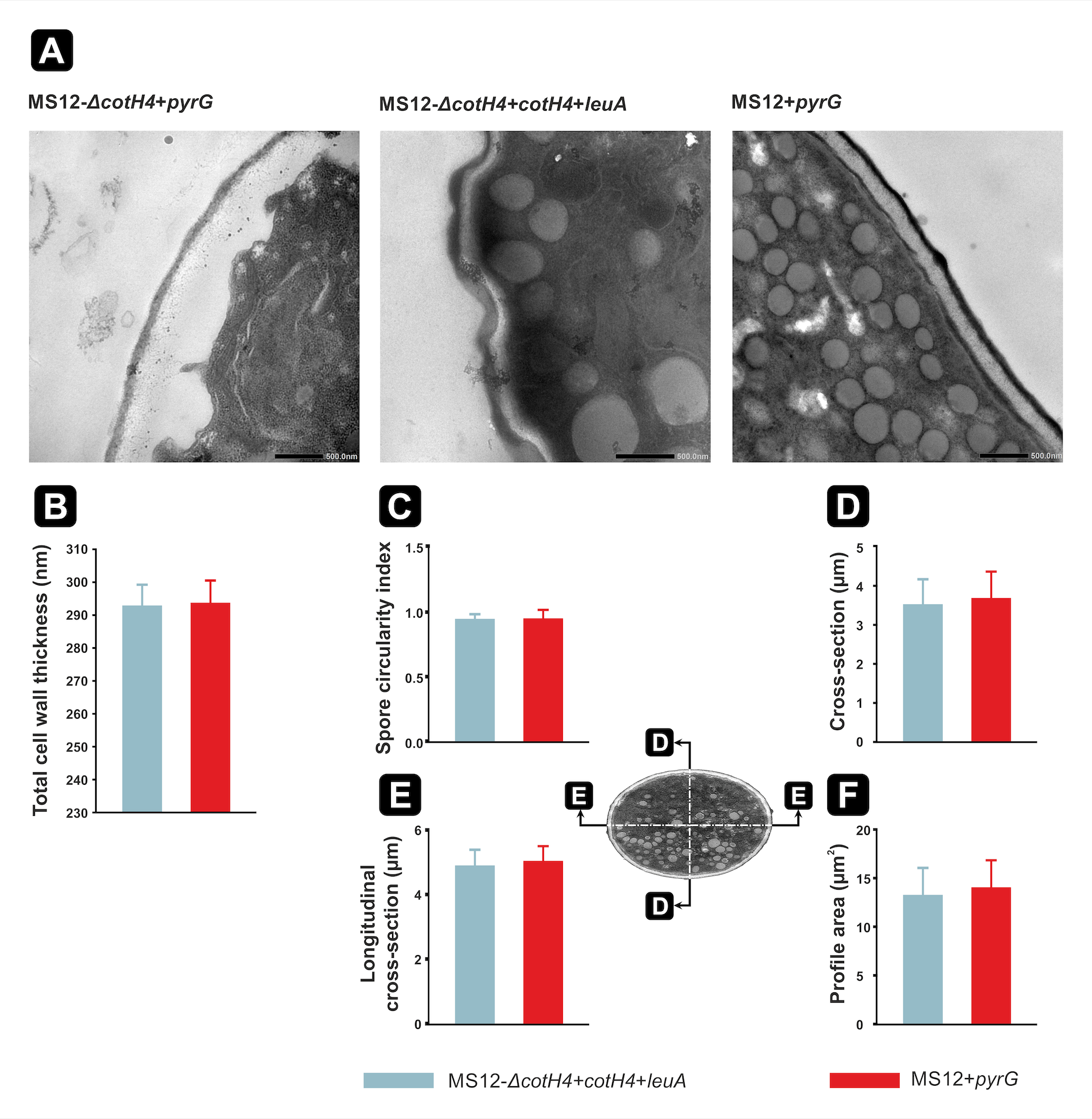

Supplement: FIG S4 [file mbio.03386-22-s0005.tif]

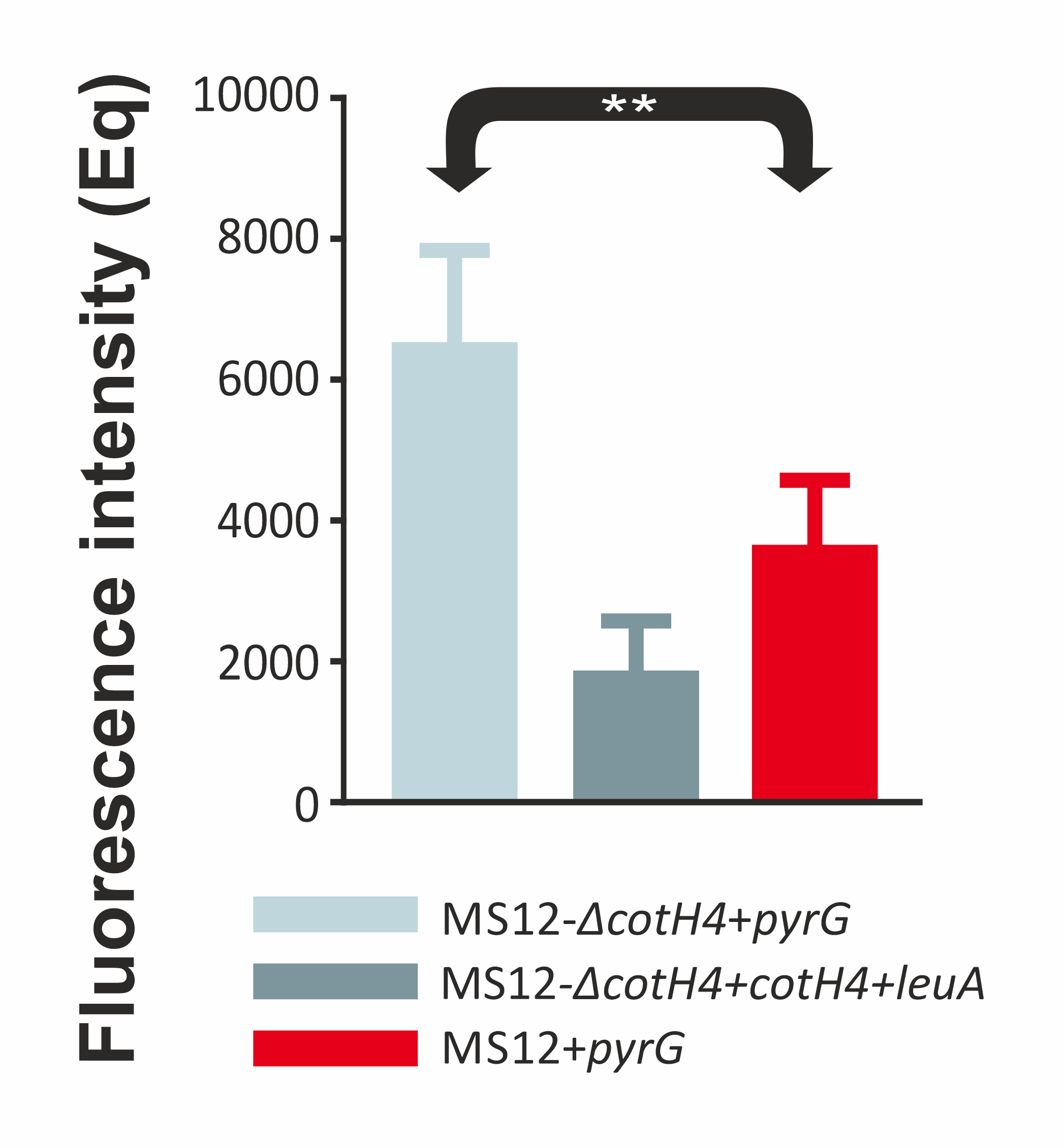

Supplement: FIG S5 [file mbio.03386-22-s0007.tif]

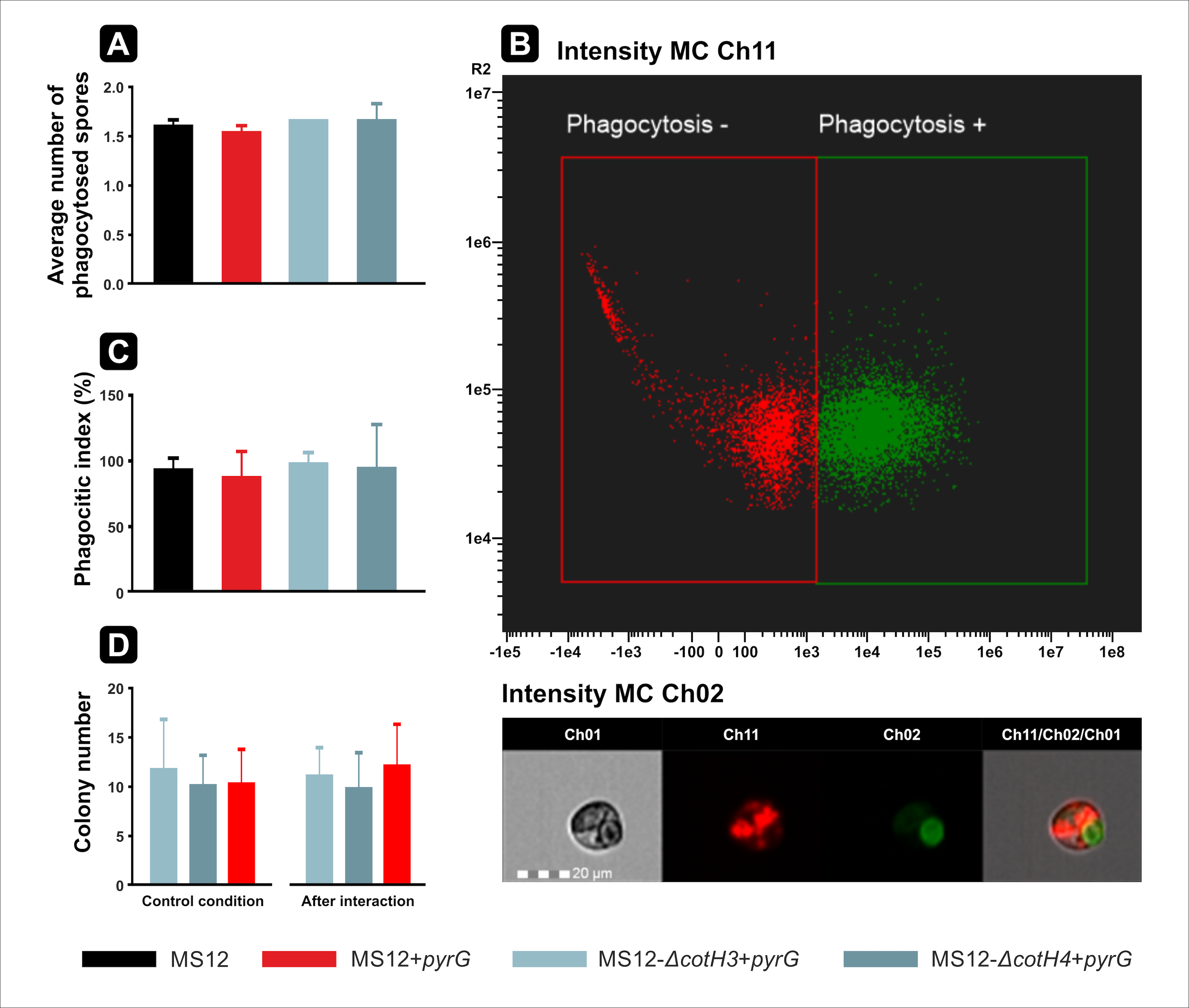

Supplement: FIG S6 [file mbio.03386-22-s0008.tif]

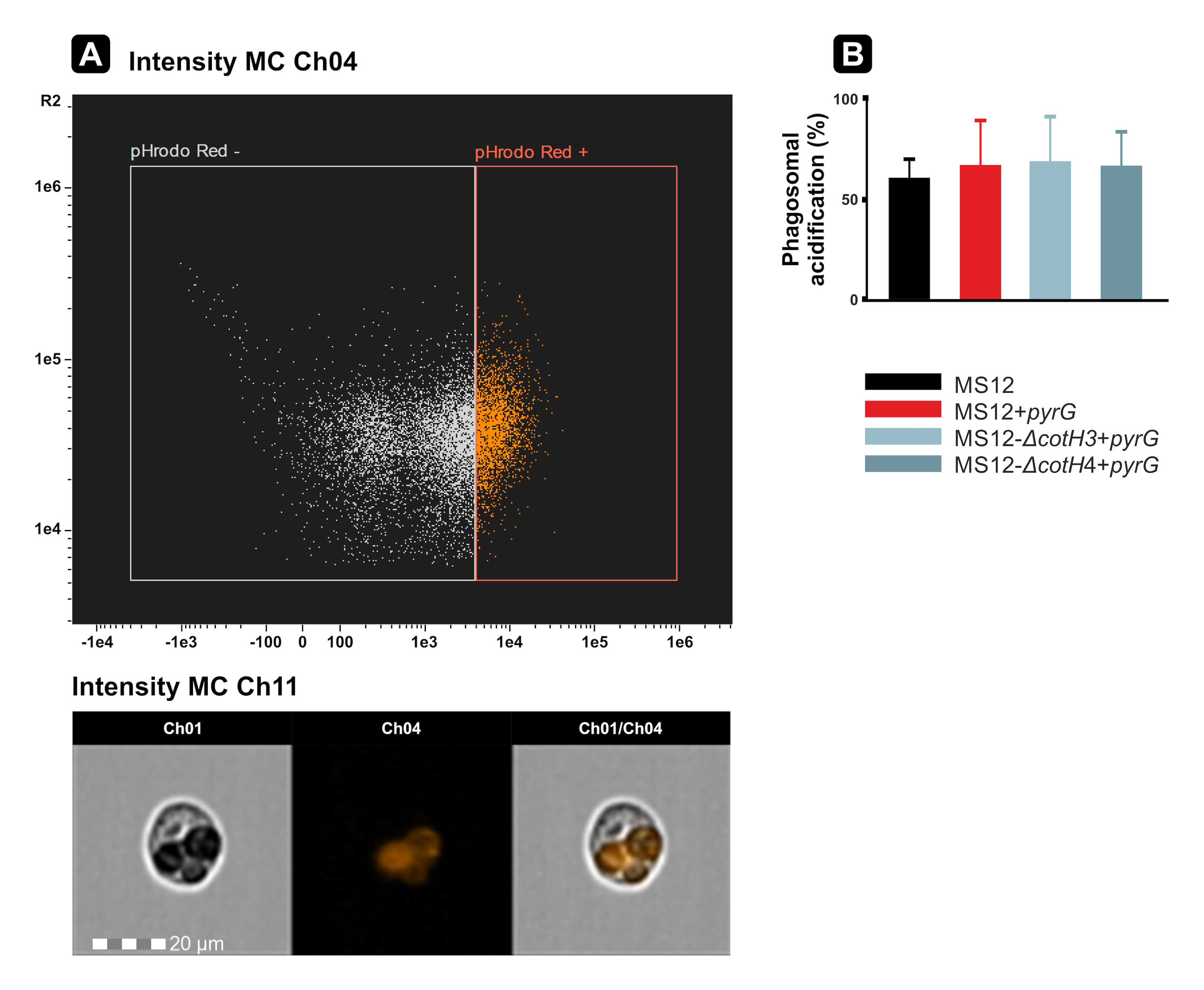

Supplement: FIG S7 [file mbio.03386-22-s0009.tif]
